# Serum sICAM-1 and Galectin-3 Levels in Diabetic Patients with COVID-19

**DOI:** 10.3390/v17071005

**Published:** 2025-07-17

**Authors:** Busra Karahan, Dogan Nasir Binici, Omer Karasahin, Sibel İba Yilmaz, Ahmet Kiziltunc, Filiz Mercantepe

**Affiliations:** 1 Department of Internal Medicine, Erzurum Regional Training and Research Hospital, Erzurum 25240, Türkiye; busra.karahan@saglik.gov.tr (B.K.); drdnb@hotmail.com (D.N.B.); 2 Department of Infectious Diseases and Clinical Microbiology, Erzurum Regional Training and Research Hospital, Erzurum 25240, Türkiye; omer.karasahin@sbu.edu.tr (O.K.); dr.syilmaz@windowslive.com (S.İ.Y.); 3 Department of Medical Biochemistry, Atatürk University School of Medicine, Erzurum 25240, Türkiye; akiziltunc@atauni.edu.tr; 4 Department of Endocrinology and Metabolism, Faculty of Medicine, Recep Tayyip Erdogan University, Rize 53100, Türkiye

**Keywords:** coronavirus disease, diabetes mellitus, sICAM-1, galectin-3

## Abstract

Introduction: This study aimed to evaluate the diagnostic and prognostic value of soluble intercellular adhesion molecule-1 (sICAM-1) and galectin-3 in patients with type 2 diabetes mellitus (T2D) diagnosed with coronavirus disease 2019 (COVID-19). Participants and Method: This prospective observational study included 45 adult patients (≥18 years) with T2D and confirmed COVID-19 who were followed in the Infectious Diseases and Clinical Microbiology departments between May and June 2022. The control group consisted of 45 healthy volunteers without chronic illness who were presented to the internal medicine outpatient clinic. In addition to routine laboratory biomarkers assessed at hospital admission, the serum levels of sICAM-1 and galectin-3 were measured via ELISA kits. Results: The median age of the patients was 66 years (range: 41–77), and 23 (51.1%) were male. Hypertension was the most common comorbidity in addition to diabetes. Compared with those in the control group, the serum levels of both galectin-3 and sICAM-1 were significantly elevated in patients with COVID-19 and T2D (*p* < 0.001). However, there was no significant difference in galectin-3 or sICAM-1 levels between survivors and nonsurvivors (*p* = 0.240 and *p* = 0.266, respectively). Conclusion: Galectin-3 and sICAM-1 demonstrated stronger diagnostic utility than conventional biomarkers in T2D patients with COVID-19. The elevated levels of these markers may reflect the underlying systemic inflammation observed in diabetic patients with COVID-19. The strong correlation between galectin-3 and sICAM-1 suggests a potential link in their inflammatory regulation, although causality cannot be inferred.

## 1. Introduction

Diabetes mellitus (DM) is a chronic metabolic disorder characterized by persistent hyperglycemia resulting from impaired insulin secretion, insufficient insulin production, or decreased insulin sensitivity [1]. Individuals with diabetes are more susceptible to infections due to immune dysfunction. Notably, coronavirus disease 2019 (COVID-19) exacerbates severe diabetic complications, including diabetic ketoacidosis, a hyperosmolar hyperglycemic state, and profound insulin resistance [2,3]. Several mechanisms have been proposed to explain the more severe course of COVID-19 in diabetic patients. These include chronic hyperglycemia, insulin resistance-associated systemic inflammation, increased expression of cellular adhesion molecules facilitating viral entry, impaired T-cell-mediated immunity, and an increased propensity for hyperinflammation and cytokine storms [4]. Furthermore, in the context of diabetes, the endothelial expression of vascular and intercellular adhesion molecules is upregulated, promoting leukocyte adhesion to the vascular endothelium and initiating the inflammatory cascade [5].

Galectin-3 is a β-galactoside-binding lectin secreted by activated macrophages, neutrophils, and epithelial cells of the gastrointestinal and respiratory tracts [6,7]. During viral infections, galectin-3 may act as a viral attachment factor, facilitating entry into immune cells [8]. Angiotensin-converting enzyme 2 (ACE2), a key receptor for SARS-CoV-2, mediates viral entry into host cells [9]. Galectin-3 has been shown to bind to ACE2 and to exhibit structural similarity to the SARS-CoV-2 spike protein, suggesting a potential role in viral pathogenesis. Moreover, galectin-3 can promote the release of proinflammatory cytokines (e.g., IL-1, IL-6, and TNF-α) and adhesion molecules (e.g., ICAM-1 and VCAM-1), contributing to the development of cytokine storm syndrome [10]. Elevated galectin-3 levels have also been linked to systemic inflammation and fibrosis [11].

Soluble intercellular adhesion molecule-1 (sICAM-1) is released from endothelial cells in response to inflammatory cytokines. It binds to leukocyte integrins, enabling firm adhesion to the endothelium and subsequent transmigration into surrounding tissues, where leukocytes contribute to inflammation through cytokine production and phagocytic activity [12]. In the context of COVID-19, overproduction of proinflammatory cytokines contributes to endothelial injury, vascular leakage, and tissue damage, phenomena collectively known as the cytokine storm. Elevated circulating levels of sICAM-1 have been associated with endothelial dysfunction in this context [13]. The detection of adhesion molecules such as sICAM-1 at the site of inflammation or in systemic circulation is thus considered a marker of disease pathogenesis [14].

The aim of this study was to explore the serum levels of sICAM-1 and galectin-3 in patients with type 2 diabetes mellitus and COVID-19 and compare them with those in healthy individuals to provide insight into the inflammatory processes associated with this comorbidity.

## 2. Participants and Methods

This prospective observational study was conducted between May and June 2022 and included 45 hospitalized patients with confirmed COVID-19 and type 2 diabetes mellitus (T2DM) who were followed in the Infectious Diseases and Clinical Microbiology departments. The control group consisted of 45 healthy volunteers without any known chronic diseases who presented to the internal medicine outpatient clinic for routine evaluations.

COVID-19 diagnosis was based on clinical and radiological findings, supported by a positive result for SARS-CoV-2 obtained from upper respiratory tract specimens via real-time reverse transcriptase–polymerase chain reaction (RT–PCR) performed in laboratories authorized by the Turkish Ministry of Health.

The inclusion criteria were age ≥ 18 years, RT–PCR-confirmed COVID-19 diagnosis, known history of T2DM, and hospitalization for COVID-19 treatment. The diagnosis of T2DM was based on the American Diabetes Association (ADA) criteria, including fasting plasma glucose ≥ 126 mg/dL, 2 h plasma glucose ≥ 200 mg/dL during OGTT, HbA1c ≥ 6.5%, or random plasma glucose ≥ 200 mg/dL with classic symptoms of hyperglycemia [15]. The exclusion criteria included age under 18 years, negative PCR results despite suggestive imaging findings, absence of a confirmed COVID-19 diagnosis, and nondiabetic individuals.

After enrollment, patients were further stratified into two subgroups on the basis of in-hospital mortality: deceased patients and surviving patients.

Blood samples were obtained from all participants at the time of hospital admission, prior to the initiation of treatment. Serum samples were collected in gel-containing vacutainer tubes and centrifuged at 4000 rpm for 10 min. The separated sera were aliquoted into Eppendorf tubes and stored for further analysis.

Serum levels of sICAM-1 and galectin-3 were measured using commercial sandwich human ELISA kits (Sunlong Biotech, Hangzhou, China); catalogue numbers: SL2065Hu (LOT:20211112) for galectin-3 and SL1607Hu (LOT:20211112) for sICAM-1, following the manufacturer’s instructions. Each assay included both positive and negative control wells provided in the kit to validate assay performance. The absorbance was read at 450 nm using a microplate reader. Results were calculated based on standard curves. The manufacturer does not provide diagnostic cut-off values; instead, optimal thresholds were identified via receiver operating characteristic (ROC) curve analysis in the current study (see Section 3).

The study was approved by the Local Ethics Committee of Regional Training and Research Hospital (approval date: 18 April 2022, approval number: 2022/05-45) and was conducted in accordance with the principles of the Declaration of Helsinki. Written informed consent was obtained from all participants. In cases where patients were unable to provide consent owing to their clinical condition, informed consent was obtained from their legal representatives.

### Statistical Analysis

The normality of the distribution of quantitative variables was assessed via the Kolmogorov–Smirnov test (*p* > 0.50). The chi-square test or Fisher’s exact test was applied for categorical variables. Continuous variables were compared between the mortality and nonmortality groups and between the patient and control groups via Student’s *t* test or the Mann–Whitney U test, as appropriate. Relationships between biomarkers were determined via Pearson’s correlation test. Coefficients of 0.2–0.39 were interpreted as indicating weak correlations, 0.4–0.59 moderate correlations, 0.6–0.79 strong correlations, and 0.8–1 very strong correlations. A receiver operating characteristic (ROC) curve was constructed to determine the predictive ability of the biomarkers used for the diagnosis of COVID-19. The Youden index was used to determine the optimal cut-off point.

## 3. Results

### 3.1. Baseline Characteristics of the Patient Group

The study included 45 hospitalized patients with type 2 diabetes mellitus (T2DM) and confirmed COVID-19 and 45 healthy control subjects. The median age of the patients was 66 years (range: 41–77), and 51.1% were male. All patients were hypoxic at admission, and 31.1% exhibited tachypnea. The most common presenting symptoms were dyspnea, cough, and general malaise. Hypertension was the most common comorbidity, with the exception of diabetes. Baseline clinical characteristics are presented in Table 1.

### 3.2. Comparison of Biomarker Levels Between Patients and Healthy Controls

**Figure 1:**
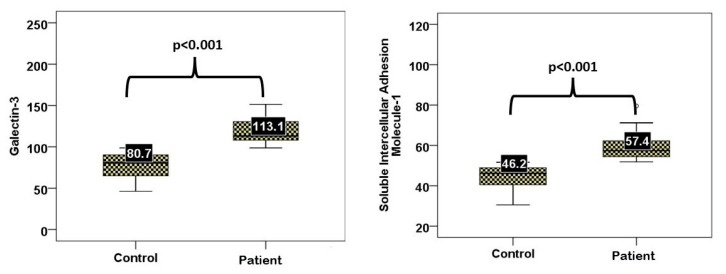
Serum sICAM-1 and galectin-3 levels in patients versus controls.

The serum levels of galectin-3 and sICAM-1 were significantly greater in the diabetic COVID-19 group than in the healthy control group (*p* < 0.001 for both). The median galectin-3 level was 113.1 ng/mL in patients and 80.7 ng/mL in controls; the median sICAM-1 level was 57.4 ng/mL in patients and 46.2 ng/mL in controls (Figure 1).

Several routine laboratory markers also differed significantly between the groups. The leukocyte count, neutrophil percentage, HbA1c, glucose, LDH, BUN, creatinine, D-dimer, CRP, ferritin, and fibrinogen were significantly greater in the patient group. In contrast, the platelet count, lymphocyte percentage, and hemoglobin, total cholesterol, LDL-C, HDL-C, albumin, creatine kinase, and total bilirubin levels were significantly lower (Table 2).

### 3.3. Diagnostic Performance of Galectin-3 and sICAM-1

**Figure 2:**
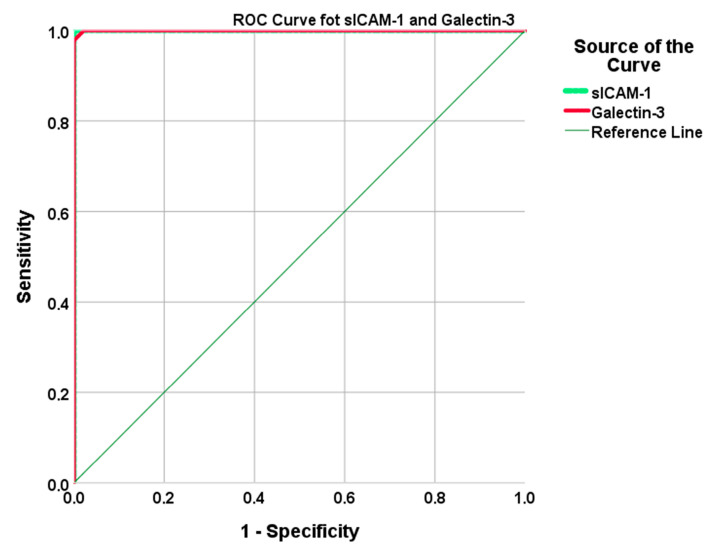
The ROC curves demonstrate the diagnostic performance of serum sICAM-1 and galectin-3 levels in differentiating diabetic COVID-19 patients from healthy controls. (Galectin-3: AUC = 1.000, cut-off ≥ 98.16 ng/mL, sensitivity = 100%, specificity = 97.8%, sICAM-1: AUC = 1.000, cut-off ≥ 51.76 ng/mL, sensitivity = 100%, specificity = 100%).

As shown in Figure 2, both galectin-3 and sICAM-1 demonstrated high diagnostic performance based on ROC analysis. Galectin-3 ≥ 98.16 ng/mL and sICAM-1 ≥ 51.76 ng/mL both showed high sensitivity and specificity in differentiating diabetic COVID-19 patients from healthy individuals (Table 3).

### 3.4. Correlation Analysis

**Figure 3:**
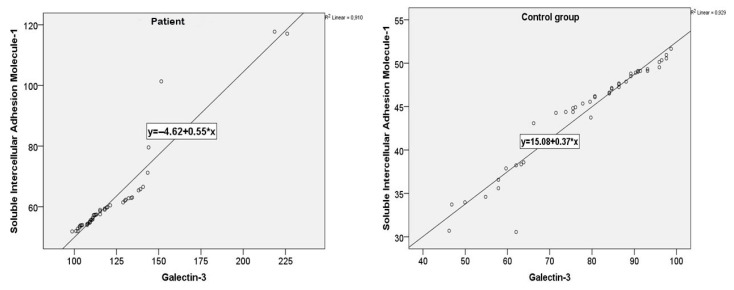
sICAM-1 and galectin-3 value correlations for patients and the control group.

A very strong and statistically significant positive correlation was observed between the serum sICAM-1 and galectin-3 levels (r = 0.954, *p* < 0.001). Mild positive correlations were also identified between both biomarkers and triglyceride levels (sICAM-1: r = 0.373, *p* = 0.012; galectin-3: r = 0.400, *p* = 0.006). The same strong correlation between sICAM-1 and galectin-3 was confirmed in both the patient and control groups (Figure 3).

### 3.5. Subgroup Analysis of Galectin-3 and ICAM-1 Levels Based on Glycemic Status and Clinical Symptoms

In subgroup analysis, both galectin-3 and sICAM-1 levels showed significant variation according to glycemic status and clinical presentation. Patients with elevated HbA1c (≥6.45) and hyperglycemia (glucose ≥ 120 mg/dL) demonstrated the highest median values of galectin-3 (115.27 ng/mL [IQR: 109.23–132.13]) and sICAM-1 (57.53 ng/mL [IQR: 54.76–62.79]). Among clinical features, galectin-3 and sICAM-1 levels were notably higher in those with malaise (*p* = 0.017 for both) and myalgia (*p* = 0.005 and *p* = 0.004, respectively). While fever and sore throat were also associated with higher biomarker levels, these did not reach statistical significance (*p* = 0.09 and *p* = 0.056, respectively). These findings are summarized in Table 4.

### 3.6. Biomarker Distribution According to Mortality Status

When stratified by in-hospital mortality, no significant differences were found in galectin-3 or sICAM-1 levels between survivors and nonsurvivors (Table 5). However, the platelet count and lymphocyte percentage were significantly lower in patients who died, whereas the neutrophil percentage and international normalized ratio (INR) were significantly greater.

## 4. Discussion

This study evaluated serum galectin-3 and sICAM-1 levels in patients with type 2 diabetes mellitus (T2DM) who were hospitalized with COVID-19 and compared them to those in healthy controls. The main findings indicate that both biomarkers were significantly elevated in the patient group and exhibited a strong positive correlation with one another. Although the study does not permit causal inference or clinical prediction due to its cross-sectional design, these results offer descriptive insight into the inflammatory profile associated with the dual burden of diabetes and COVID-19.

Galectin-3 is a multifunctional lectin involved in immune cell activation, cytokine release, and fibrosis. It has been implicated in the pathogenesis of both diabetes and viral infections, including SARS-CoV-2. Prior studies have yielded conflicting results regarding galectin-3 levels in individuals with diabetes, with some reporting increased levels and others reporting reductions or no significant differences [16,17,18,19]. However, more consistent findings have emerged in COVID-19 patients, where galectin-3 has been shown to be correlated with disease severity and inflammatory burden [20,21,22]. The elevated galectin-3 levels observed in our cohort may reflect the combined influence of underlying metabolic dysfunction and acute viral inflammation, although the individual contributions cannot be distinguished in the absence of disease-specific control groups.

sICAM-1, an adhesion molecule released from activated endothelial cells, plays a key role in leukocyte trafficking and endothelial dysfunction [23]. Like galectin-3, sICAM-1 has been studied in both patients with diabetes and those with COVID-19, but findings have varied depending on disease severity, glycemic control, and the presence of complications [24,25,26]. One study reported significantly higher sICAM-1 levels than did a control group, and that sICAM-1 was positively correlated with fasting and postprandial blood sugar [27]. In the context of COVID-19, sICAM-1 elevation has been associated with cytokine storm and vascular injury [28,29,30,31], which may explain the increased levels observed in diabetic COVID-19 patients. Subgroup analyses in our study supported these observations by demonstrating that both galectin-3 and sICAM-1 levels were highest in patients with poor glycemic control (HbA1c ≥ 6.45) and hyperglycemia (glucose ≥ 120 mg/dL). Furthermore, these biomarkers were significantly elevated in individuals presenting with systemic symptoms such as malaise and myalgia, suggesting a more pronounced inflammatory response in these subgroups.

The strong positive correlation between galectin-3 and sICAM-1 in both the patient and control groups suggests a potentially shared regulatory mechanism, possibly mediated by proinflammatory cytokines such as IL-1, IL-6, TNF-α, and IFN-γ, which are known to be upregulated during SARS-CoV-2 infection [10,14,32]. Moreover, prior evidence supports a role for galectin-3 in the upregulation of ICAM-1 and other adhesion molecules, providing a biological rationale for their coelevation [33].

In addition, we observed mild positive correlations between serum triglyceride levels and both galectin-3 and sICAM-1. While these associations were not the primary focus of this study, they align with previous reports suggesting that postprandial metabolic status and insulin resistance may influence endothelial and immune activation markers [34,35,36,37,38]. A positive correlation has been shown between sICAM and postprandial triglycerides and insulin after a rich diet in healthy individuals [34]. In another study, an increase in sICAM-1 was observed following the oral glucose tolerance test. No correlation was found between isolated hyperlipidemia and sICAM-1. In light of these findings, it has been reported that glucose metabolism and insulin resistance, but not hyperlipidemia in satiety, cause an increase in sICAM-1 levels [35]. More than one study in the literature has detected a positive correlation between triglyceride and both sICAM-1 and galectin-3 values [34,35,36,37,38]. Similarly, in the present study, triglyceride values were mildly positively correlated with both sICAM-1 and galectin-3.

Although both biomarkers demonstrated high diagnostic accuracy on the basis of ROC analysis, these findings should be viewed as exploration. Since our study lacked disease-specific control groups, such as patients with T2DM alone or COVID-19 alone, we cannot determine whether the observed elevations in galectin-3 and sICAM-1 are specific to the combination of these two conditions or primarily driven by one of them. This limits the specificity of our findings as potential diagnostic biomarkers. The absence of disease control groups (e.g., patients with COVID-19 without diabetes or vice versa) and the small sample size limit the generalizability and clinical applicability of our results. Nevertheless, the data presented here contributes to the growing body of literature exploring biomarker dynamics in COVID-19 and highlights the need for more robust studies in metabolically vulnerable populations.

### 4.1. Limitations

This study has several limitations that should be acknowledged. First, the cross-sectional design precludes causal inference or prognostic evaluation. Although patients were stratified by mortality status, the small number of fatal cases limited the power to detect differences in biomarker levels related to clinical outcomes. Second, the control group consisted only of healthy individuals without diabetes or COVID-19. The absence of disease-specific control groups precludes differentiation between the individual and combined effects of diabetes and COVID-19 on biomarker levels. Third, although ROC analysis was performed, the lack of an independent validation cohort limits the generalizability of the diagnostic accuracy findings. Finally, no longitudinal follow-up was available to assess the predictive value of these biomarkers over the disease course. While the observed correlation between galectin-3 and sICAM-1 supports a potential biological relationship, no mechanistic experiments were conducted in this study. Therefore, the proposed involvement of galectin-3 in inducing sICAM-1 release via cytokine pathways remains hypothetical and warrants further investigation.

### 4.2. Future Directions

Future studies should aim to include larger and more diverse cohorts with well-defined control groups, including patients with diabetes but without COVID-19 and patients with COVID-19 without diabetes. Longitudinal sampling and follow-up data will also be critical for evaluating the prognostic potential of galectin-3 and sICAM-1. In addition, experimental studies are needed to clarify the mechanistic relationship between these two biomarkers and their role in the immune–inflammatory axis of COVID-19 in diabetic patients. These efforts will contribute to a more comprehensive understanding of their clinical utility and pathophysiological relevance.

## 5. Conclusions

This study provides preliminary observational data on the serum levels of galectin-3 and sICAM-1 in diabetic patients hospitalized with COVID-19. Both biomarkers were significantly elevated compared with those in healthy individuals and exhibited strong correlations, suggesting a shared inflammatory response mechanism. While these findings highlight their potential as inflammatory markers in this high-risk group, the results should be interpreted as hypothesis-generating rather than conclusive. Further studies with more rigorous designs are needed to determine the diagnostic or prognostic utility of galectin-3 and sICAM-1 in clinical practice.

## Figures and Tables

**Table 1:** Baseline characteristics of the patient group.

	Total (*n* = 45)
Demographic Findings	
Age (years), median (range)	66 (41–77)
Sex (male), *n* (%)	23 (51.1)
Smokers, *n* (%)	13 (28.9)
Comorbid Diseases, *n* (%)	
Hypertension	37 (82.2)
Nondiabetes endocrine disease	26 (57.8)
Coronary artery disease	20 (44.4)
Chronic obstructive pulmonary disease	12 (26.7)
Heart failure	8 (17.8)
Diabetic neuropathy	7 (15.6)
Chronic kidney failure	4 (8.9)
Asthma	3 (6.7)
Chronic liver disease	1 (2.2)
Clinical Symptoms, *n* (%)	
Shortness of breath	38 (84.4)
Cough	31 (68.9)
Malaise	15 (33.3)
Fever	11 (24.4)
Headache	3 (6.7)
Sore throat	3 (6.7)
Myalgia	1 (2.3)
Nausea-vomiting	1 (2.3)
Vital Findings, *n* (%)	
Hypoxia (oxygen saturation ≤ 93%)	45 (100)
Tachypnea (>100 beats/min)	14 (31.1)
Tachycardia (>30 respiration rate/min)	7 (1.6)
Fever (≥38.3 °C)	5 (11.1)
Hypotension (≤90/60 mm/hg)	1 (2.2)

**Table 2:** A comparison of biomarkers at the time of presentation to the hospital with those in the control group.

	Control Group (*n* = 45)	COVID-19 (*n* = 45)	*p* *
Galectin-3 (ng/mL)	80.67 (46.20–98.72)	113.08 (98.72–226.06)	<0.001
sICAM-1 (ng/mL)	46.19 (30.56–51.66)	57.42 (51.86–117.72)	<0.001
Platelet count (10^3^/L)	268 (158–466)	243 (11–481)	0.03
Leukocyte count (10^3^/L)	7.55 (3.91–12.74)	8.84 (1.56–18.80)	0.002
Lymphocyte percentage (%)	29.7 (0–51.5)	14.7 (1–45.7)	<0.001
Neutrophil percentage (%)	59 (37.9–94.5)	78.8 (34.8–97.7)	<0.001
Hemoglobin (g/dL)	14.6 (9.1–18.6)	13.9 (7.2–16.9)	0.043
Hemoglobin A1c (mmol/mol)	5.7 (4.6–6.4)	8.8 (6.5–14.6)	<0.001
Total cholesterol (mg/dL)	194 (133–303)	156 (86–241)	<0.001
Low-density lipoprotein cholesterol (mg/dL)	127 (64–223)	99 (50–204)	0.004
High-density lipoprotein cholesterol (mg/dL)	49.4 (32.1–80.9)	38.8 (16–68)	<0.001
Triglyceride (mg/dL)	118 (54–418)	151 (24–548)	0.205
Glucose (mg/dL)	93 (70–165)	240 (103–673)	<0.001
ALT (U/L)	21 (9–71)	20 (4–199)	0.756
AST (U/L)	19 (6–72)	22 (8–321)	0.756
LDH (U/L)	193 (51–309)	296 (179–1165)	<0.001
Albumin (g/L)	45 (37–53)	40 (24–48)	<0.001
Creatine kinase (U/L)	90 (39–272)	61 (14–706)	0.036
BUN (mg/dL)	16 (9–48)	20 (6–150)	0.01
Creatinine (mg/dL)	0.85 (0.43–1.41)	1 (0.44–8.2)	0.006
Total bilirubin (mg/dL)	0.8 (0.3–2.1)	0.5 (0.2–1.8)	<0.001
Direct bilirubin (mg/dL)	0.2 (0.1–0.7)	0.2 (0.1–0.7)	0.107
D-dimer (ng/mL)	406 (190–2899)	858 (190–35,000)	<0.001
CRP (mg/L)	2.3 (0.1–151.0)	61.3 (4.3–368.0)	<0.001
Ferritin (ng/mL)	45 (3–350)	495 (51–44,725)	<0.001
B12 vitamin (pg/mL)	317 (193–628)	352 (115–1792)	0.511
INR	1.00 (0.90–1.66)	1.00 (0.90–3.89)	0.997
PT (s)	14.1 (12.2–21.9)	14.1 (11.5–50.2)	0.968
APTT (s)	29.6 (25.2–67.1)	29.9 (17.6–65.0)	0.628
PTT percentage	93 (49–119)	92 (18–120)	0.920
Fibrinogen (mg/dL)	307 (136–556)	557 (306–898)	<0.001

* The Mann–Whitney U test was applied. sICAM-1: soluble intercellular adhesion molecule-1 ALT: alanine aminotransferase AST: aspartate aminotransferase, LDH: lactate dehydrogenase, BUN: blood urea nitrogen, CRP: C-reactive protein, INR: international normalized ratio, PT: prothrombin time, PTT: partial thromboplastin time, APTT: activated partial thromboplastin time.

**Table 3:** The diagnostic value, sensitivity, and specificity of the biomarkers at the time of presentation in diabetic patients with COVID-19.

Biomarker	AUC (95% CI)	Cut-Off	Specificity	Sensitivity	*p*
Galectin-3 ng/mL	1.000 (0.999–1.000)	≥98.16	97.8%	100%	<0.001
sICAM-1 ng/mL	1.000 (1.000–1.000)	≥51.76	100%	100%	<0.001
Platelet count (10^3^/L)	0.633 (0.517–0.749)	≤204.500	40.0%	91.1%	0.030
Leukocyte count (10^3^/L)	0.689 (0.579–0.800)	≥8.420	60.0%	75.6%	0.002
Lymphocyte percentage (%)	0.831 (0.738–0.924)	≤23.25	80.0%	86.7%	<0.001
Neutrophil percentage (%)	0.830 (0.737–0.923)	≥70.25	77.8%	86.7%	<0.001
Hemoglobin (g/dL)	0.624 (0.508–0.740)	≤15.00	75.6%	46.7%	0.043
HbA1c	1.000 (1.000–1.000)	≥6.45	100%	100%	<0.001
Total cholesterol (mg/dL)	0.740 (0.635–0.845)	≤176.5	66.7%	75.6%	<0.001
Low-density lipoprotein cholesterol (mg/dL)	0.676 (0.562–0.79)	≤87.5	46.7%	88.9%	0.004
High-density lipoprotein cholesterol (mg/dL)	0.815 (0.729–0.901)	≤40.5	64.4%	88.9%	<0.001
Glucose (mg/dL)	0.980 (0.957–1.000)	≥120	95.6%	91.1%	<0.001
LDH (U/L)	0.907 (0.847–0.967)	≥235.5	88.9%	82.2%	<0.001
Albumin (g/L)	0.829 (0.741–0.917)	≤41.5	73.3%	88.9%	<0.001
Creatine kinase (U/L)	0.629 (0.507–0.750)	≤56.5	46.7%	86.7%	0.036
BUN (mg/dL)	0.659 (0.545–0.772)	≥22.5	82.2%	46.7%	0.010
Creatinine (mg/dL)	0.669 (0.556–0.782)	≥1.005	91.1%	46.7%	0.006
Total bilirubin (mg/dL)	0.739 (0.637–0.841)	≤0.52	53.3%	88.9%	<0.001
D-dimer (ng/mL)	0.742 (0.638–0.846)	≥583	73.3%	75.6%	<0.001
CRP (mg/L)	0.944 (0.893–0.995)	≥11	86.7%	91.1%	<0.001
Ferritin (ng/mL)	0.953 (0.914–0.991)	≥189	95.6%	84.4%	<0.001
Fibrinogen (mg/dL)	0.955 (0.916–0.994)	≥431	95.6%	86.7%	<0.001

Abbreviations: sICAM: soluble intercellular adhesion molecule-1, HbA1c: hemoglobin A1c, LDH: lactate dehydrogenase, BUN: blood urea nitrogen, CRP: C-reactive protein.

**Table 4:** Galectin-3 and ICAM-1 according to glycemic subgroups and clinical symptoms.

Subgroup/Symptom	Galectin-3 Median (IQR)—Present	Absent	*p*-Value	ICAM-1 Median (IQR)—Present	Absent	*p*-Value
HbA1c < 6.45/Glucose < 120 mg/dL (*n* = 43)	80.68 (64.42–89.76)			46.20 (39.55–48.86)		
HbA1c < 6.45/Glucose ≥ 120 mg/dL (*n* = 2)	89.12 (84.33–93.92)			48.61 (47.09–50.14)		
HbA1c ≥ 6.45/Glucose < 120 (*n* = 4)	109.51 (106.20–112.39)			55.75 (53.69–57.55)		
HbA1c ≥ 6.45/Glucose ≥ 120 mg/dL (*n* = 41)	115.27 (109.23–132.13)			57.53 (54.76–62.79)		
Fever	133.75 (108.68–147.74)	112.81 (105.92–120.87)	0.090	62.88 (54.66–90.45)	57.38 (54.17–60.31)	0.090
Cough	111.98 (107.58–125.08)	115.27 (111.02–133.34)	0.339	57.33 (54.12–60.94)	58.75 (56.57–62.88)	0.309
Sore throat	139.14 (134.01–139.95)	112.26 (107.72–127.79)	0.056	65.79 (63.61–66.17)	57.33 (54.32–61.62)	0.056
Headache	113.08 (112.81–128.26)	113.63 (107.72–130.37)	0.439	57.43 (57.38–64.32)	57.43 (54.32–62.23)	0.453
Malaise	130.50 (112.25–141.29)	111.16 (105.51–118.82)	0.017	62.30 (56.19–68.50)	56.79 (53.97–59.60)	0.017
Myalgia	134.82 (119.78–143.58)	110.88 (107.58–119.64)	0.005	64.04 (60.09–73.30)	56.35 (53.97–59.87)	0.004

**Table 5:** Distributions of biomarkers during presentation to the hospital according to mortality status.

	Mortality		
	None (*n* = 38)	Present (*n* = 7)	*p*
Galectin-3 ng/mL	115.27 (98.71–226.05)	109.23 (103.16–134.28)	0.240
sICAM-1 ng/mL	58.01 (51.86–117.72)	55.16 (53.16–63.07)	0.266
Platelet count (10^3^/L)	247 (116–481)	164 (110–391)	0.042
Leukocyte count (10^3^/L)	8.86 (3.73–18.50)	8.73 (1.56–18.80)	0.742
Lymphocyte percentage (%)	15.1 (2.3–45.7)	7 (1–22.4)	0.037
Neutrophil percentage (%)	78.05 (34.8–94.1)	88.8 (71.2–97.7)	0.017
Hemoglobin (g/dL)	14.05 (8.4–16.9)	11.6 (7.2–16.5)	0.121
HbA1c	8.55 (6.5–14.6)	9.2 (7.8–10.4)	0.500
Total cholesterol (mg/dL)	158.5 (100–240)	137 (86–241)	0.491
Low-density lipoprotein cholesterol (mg/dL)	100 (53–204)	81 (50–152)	0.247
High-density lipoprotein cholesterol (mg/dL)	38.9 (16–68)	36 (25.8–47.3)	0.742
Triglyceride (mg/dL)	137.5 (24–548)	175 (80–306)	0.280
Glucose (mg/dL)	233.5 (103–511)	284 (146–673)	0.266
ALT (U/L)	20 (8–75)	29 (4–199)	0.187
AST (U/L)	22 (9–100)	17 (8–321)	0.649
LDH (U/L)	290 (179–750)	364 (268–1165)	0.036
Albumin (g/L)	40.5 (24–48)	33 (24–38)	0.002
Creatine kinase (U/L)	61.5 (15–706)	35 (14–521)	0.253
BUN (mg/dL)	19 (6–103)	37 (12–150)	0.058
Creatinine (mg/dL)	0.92 (0.56–8.2)	1.26 (0.44–4.59)	0.301
Total bilirubin (mg/dL)	0.5 (0.2–1.8)	0.6 (0.3–0.9)	0.789
Direct bilirubin (mg/dL)	0.2 (0.1–0.7)	0.2 (0.2–0.6)	0.400
D-dimer (ng/mL)	844 (190–35.000)	1497 (442–2.404)	0.672
CRP (mg/L)	59.0 (4.3–339)	119.2 (12.6–368.0)	0.347
Ferritin (ng/mL)	450 (51–4725)	528 (159–1650)	0.594
B12 vitamin (pg/mL)	331 (115–1792)	405 (279–822)	0.133
INR	1.00 (0.90–1.69)	1.27 (1.00–3.89)	0.023
PT (s)	14.05 (11.50–22.30)	16.90 (13.40–50.20)	0.056
APTT (s)	29.85 (26.00–41.20)	30.00 (17.60–65.00)	0.826
PTT percentage	92.5 (48.0–120.0)	70.0 (18.0–100.0)	0.056
Fibrinogen (mg/dL)	561 (306–898)	504 (370–717)	0.491

Abbreviations: sICAM-1: soluble intercellular adhesion molecule-1, ALT: alanine aminotransferase, AST: aspartate aminotransferase, LDH: lactate dehydrogenase, BUN: blood urea nitrogen, CRP: C-reactive protein, INR: international normalized ratio, PT: prothrombin time, PTT: partial thromboplastin time, APTT: activated partial thromboplastin time.

## Data Availability

All data generated or analyzed during this study are included in this article. The data will be available upon reasonable request (contact filiz.mercantepe@saglik.gov.tr).
